# Global action on antimicrobial resistance: lessons from the history of climate change and tobacco control policy

**DOI:** 10.1136/bmjgh-2022-009283

**Published:** 2022-07-28

**Authors:** Emma Pitchforth, Elta Smith, Jirka Taylor, Sally Davies, Gemma-Claire Ali, Camilla d’Angelo

**Affiliations:** 1 College of Medicine and Health, University of Exeter, Exeter, UK; 2 Independent Researcher, London, UK; 3 RAND Corporation, Santa Monica, California, USA; 4 United Kingdom Department of Health and Social Care, London, UK; 5 RAND Europe, Cambridge, UK

**Keywords:** health policy, public health

**Summary box**

- Antimicrobial resistance (AMR) is one of the most pressing global health challenges currently. Recognised as a complex policy issue, efforts to mitigate AMR transcend national boundaries and require global coordination.
- Some parallels have been drawn with other complex problems such as climate change and tobacco control yet few analyses have taken a comparative and historical approach to draw lessons for AMR policy.
- Practical suggestions are made for improved AMR governance globally, working on the basis of shared goals but reflecting needs and priorities at a national level.

Antimicrobial resistance (AMR) is a major threat to global public health.^1 2^ Because of AMR, drugs like antibiotics no longer work or work less well than they should. Common, easily treated infections could become life-threatening. The COVID-19 pandemic will likely worsen the AMR crisis due to increasing unnecessary antibiotic use and drug-resistant secondary infections in hospital.^3–5^

Tackling AMR requires international action across multiple sectors—including human healthcare, agriculture and the environment. Such efforts include a landmark 2015 WHO Global Action Plan on AMR, followed in 2016 by a historic United Nations (UN) Declaration on AMR and establishment of the Interagency Coordination Group on AMR. The One Health Global Leaders Group (GLG) on AMR was founded in 2020 to provide leadership and maintain political momentum on the issue. The GLG is supported by the Tripartite Joint Secretariat on AMR—a shared effort to address the One Health dimensions of AMR among the UN Food and Agriculture Organisation, World Organisation for Animal Health, WHO, and more recently, UN Environment Programme.

Lessons can be learnt from past experience. A comparative assessment of how AMR evolved as a policy problem alongside a similarly complex problem—climate change—and a relative success—tobacco control—can provide insight into how to better design future international AMR efforts and avoid problems encountered in other areas. Table 1 compares the nature of the three problems and policy actions attempted. We group lessons from our comparison into three themes: (1) how to deal with problems that feature a high degree of scientific uncertainty and complexity; (2) how to act considering an uneven global picture for decision making and (3) the critical role of social change in addressing complex problems. Although our analysis focuses on human health, reflecting the ultimate goal of AMR policy and an inevitable result of including tobacco control as a comparator, the lessons are relevant across a One Health approach (table 1).

**Table 1:** Summary of the basis of comparison between policy issues according to nature of the problem and potential policy solutions

	Antimicrobial resistance	Climate change	Tobacco control
Nature of the problem			
Significant public health consequences	+++	+++	+++
Complexity and inter-relatedness of problems	+++	+++	++
Multiple stakeholder involvement	+++	+++	++
Divergence among stakeholders	++	+++	+++
International nature of challenge that prompts need for global action	+++ Transnational	+++ Transnational	++ Multinational
Intertemporal element – time between contributing action and impact	+++	+++	+
Common resource problem	+++	+++	Does not apply
Potential policy solutions			
Cross government response requirement (progress made)	+++	+++	+++
Definitive (+++) vs amelioration (+)	+ Resistance will always occur	+++ Greenhouse gases cannot be eliminated but carbon-neutral/negative economy possible; required technology exists	+++ Potentially definitive
Nature of global action (achieved or proposed)	Achieved: Non-binding WHO Global Action Plan Proposed: Global governance mechanism	Achieved: Intergovernmental Panel on Climate Change established in 1988 United Nations Framework Convention on Climate Change established in 1992 Governance increasingly voluntary and polycentric over time	Achieved: The Framework Convention on Tobacco Control became the first and only legally binding international health treaty, adopted in 2003 Proposed: Requires governments to develop and enforce their own regulations
Extent to which policy has been able to capitalise on social change	+	++	+++

+, ++,+++ reflects relevance according to each criteria as decided by the research team and informed by literature.

## Scientific uncertainty and complexity

Decision-makers understandably want evidence of the future harms and costs of inaction. Evidence review and consolidation has long been a prerequisite for policy-making (eg, the 1964 US Surgeon General’s report on Smoking and Health).^6^ Decision-makers also require information on the benefits of taking alternative courses of action (eg, the Stern Review on climate change and O’Neill Review on AMR).

There have been increasing calls for the establishment of an ‘Intergovernmental Panel on Climate Change (IPCC) for AMR’.^7^ The IPCC provides decision-makers with scientific assessments on climate change, which can inform policy. Adopting a similar approach for AMR could be a beneficial step; the IPCC is considered to be ‘the most successful attempt in history’ to harness scientific evidence to inform policy and practice worldwide.^7^

The IPCC was designed with consensus at its core, requiring all parties to approve its published reports. This helps to ensure that IPCC outputs are widely accepted and enable policy-makers to take joint action.

The system has in practice resulted in a conservative bias in the IPCC’s official reports. New or comparatively untested evidence is less likely to be included. Steps may be taken to ensure that emerging insights or alternative views are not excluded, such as publishing submissions that do not receive consensus endorsements and broadening the range of contributors.

Appropriate and adequate surveillance, with good geographical coverage that spans One Health dimensions, is another powerful way to reduce uncertainty for AMR decision makers. The ability to capture and model key variables underpins much of the IPCC’s work. So too the capacity to obtain reliable data on indicators such as antibiotic consumption and resistance is prerequisite for effective international decision making for AMR.

## Decision making in a global context

At an international level, agreement on common, evidence-based goals is critical to successful decision making. Collective action problems, such as climate change or AMR, mean that one country’s ability to tackle the issue is affected by other countries’ actions.^8^

Access to antibiotics is also unequal. High-income countries are promoting policy measures to curb antibiotic usage, while low-income and middle-income countries are negotiating prices and availability.

Climate change and tobacco control initiatives have faced difficulties in maintaining a coordinated global approach, largely due to lack of agreement on how individual country capabilities and priorities should be reflected.^9^ The Framework Convention on Tobacco Control was largely based on approaches already implemented in several high-income countries. Action on climate change has seen a gradual disintegration of ‘global’ governance with regional and local hubs of action emerging.^10^

Both for climate change and tobacco control, common goals may be best met through individual country targets and actions based on feasibility determined at the national level.^11^ The most effective strategies may be those that focus on polycentric governance and solving specific problems in particular local contexts, and carefully adapting rather than just adopting ‘best practice’ solutions.

A global approach for bringing together evidence and analysis on AMR could also draw lessons from the IPCC’s experiences regarding the type of data collected and perspectives included. Climate change was originally characterised primarily as a technical issue, stemming from concerns voiced by climate scientists beginning in the 1980s.^10^ By the 1990s and early 2000s, climate change developed into a more widely recognised social and political issue.^10^ The social dimensions of climate change were initially underappreciated in the IPCC’s development,^12^ which limited its utility and the perceived representativeness of the evidence collected.

AMR has experienced a similar trajectory. Initially treated as a technical issue, its evolution into being viewed as a social and political challenge will be important to consider in future efforts to bring together evidence on its risks and potential harms—and using it for action and investment.

## Social change

Social change is critical to successful policy implementation yet often considered too late in the policy-making process.

Social drivers such as cooperation and conformity need to be recognised. People are more likely to do their part if they know others are doing theirs, and if their own behaviour is accountable or observable to others. Smoking bans in public places became politically attractive in part because of emerging evidence and public health campaigns highlighting the negative health impacts of passive smoking. This nurtured an appeal not to harm others, and equally a belief that others do not have the right to harm us, overriding individual objections of paternalism.

The challenges of reaching a point of self-reinforcing social norms in relation to antibiotic use may be greater than for smoking which tends to be more visible^13^ but policy-makers could consider appealing to individual rights, and to how the public’s collective responsibilities affect individual rights. This could focus on individual rights to effective drugs such as antibiotics, and more generally, to having access to the best available healthcare, which is jeopardised by the failure of others to use antibiotics appropriately.

National governments could also start valuing antimicrobials as critical infrastructure for their health systems. This approach could better integrate how to address AMR in a broader health systems context, and increase investments to ensure their availability.

Tobacco control and climate change illustrate that a high level of awareness and acceptance of the available evidence is needed to effect social change and successful policy implementation.

## Lessons for future action

A coordinated international effort is warranted to address AMR. The GLG, UN High-Level Interactive Dialogue and recent G7 summit, among other multilateral fora, provide important opportunities to agree on how this can be done.

International action requires countries to agree on shared, evidence-based goals with clear accountability and impetus to collaborate. Action can be based on shared goals but with different needs and priorities driving the course those actions take at a national level. Further consideration of how this may be operationalised to include and go beyond previously advocated models of target setting will be important.^14^

Differences stemming from an uneven global picture need to be bridged, which may involve financial and technical assistance. Agreement is paramount; UN agencies are hampered in providing such assistance without unanimous support.

There is a risk of making global architecture too complex. The GLG could serve as a platform to make supporting organisations more accountable; the GLG can advocate for Member States to more consistently and coherently monitor their own efforts and progress, and mobilise financial support. The AMR Multi Partner Trust Fund offers a financial mechanism to promote coordination within the GLG Secretariat, and provide greater support for countries in the design and implementation of One Health National Action Plans and Quadripartite Workplans. Nevertheless, the donor base consisting of the UK, Netherlands and Sweden does not represent a global effort.

There is much to do. International collective action, not just advocacy, is required to avoid the catastrophic consequences that could arise from failing to address AMR. The current pandemic serves as a sobering catalyst for change.

## Data Availability

The data from the research that helped inform this commentary is not publicly available.
